# Study of Human Papillomavirus Vaccine–Related Social Media Content Across Eight Social Media Platforms: Scoping Review of Communication Content, Information Sources, and Analytical Approaches

**DOI:** 10.2196/92040

**Published:** 2026-09-15

**Authors:** Yinghan Xu, Ruichen Cong, Mingxin Liu, Siyu Zhou, Masahiko Sakaguchi, Kayoko Katayama, Qun Jin, Shoji Nishimura, Atsushi Ogihara

**Affiliations:** 1 Graduate School of Human Sciences, Waseda University, Tokorozawa, Saitama, Japan; 2 Faculty of Human Sciences, Waseda University, 2-579-15 Mikajima, Tokorozawa, Saitama, 359-1192, Japan, 81 9041086485; 3 Department of Health Communication, Graduate School of Medicine, The University of Tokyo, Bunkyo, Tokyo, Japan; 4 School of Public Health, Hangzhou Normal University, Hangzhou, Zhejiang, China; 5 Advanced Research Center for Human Sciences, Waseda University, Tokorozawa, Saitama, Japan; 6 Department of Information Science, Faculty of Science and Technology, Kochi University, Kochi, Japan; 7 Unit of Cancer Education, Kanagawa Cancer Center Research Institute, Yokohama, Kanagawa, Japan; 8 Graduate School of Informatics, Gunma University, Maebashi, Gunma, Japan

**Keywords:** cervical neoplasms, health communication, information dissemination, papillomavirus vaccines, social media, HPV vaccine, vaccine misinformation, digital health, eHealth, mHealth, infodemiology, content analysis, scoping review, mobile health

## Abstract

**Background:**

Social media has become an important channel for health information dissemination and public discussion of human papillomavirus (HPV) vaccination. Previous reviews have examined social media and HPV vaccination, often focusing on single platforms, broader HPV-related topics, or the potential effects on knowledge, leaving limited cross-platform synthesis specifically focused on HPV vaccine–related communication content, information sources, and analytical approaches.

**Objective:**

This scoping review aimed to map and synthesize peer-reviewed studies examining HPV vaccine–related social media content, with particular attention to communication content, information sources, and methodological characteristics.

**Methods:**

Following the PRISMA-ScR (Preferred Reporting Items for Systematic Reviews and Meta-Analyses extension for Scoping Reviews) guidelines, we searched PubMed, Web of Science, MEDLINE, Embase, and Scopus on June 10, 2026. Backward and forward citation searches were completed on July 17, 2026. We included peer-reviewed English-language original studies published between 2006 and 2026 that analyzed HPV vaccine–related communication content on social media. Data were charted using a structured extraction form and synthesized descriptively. No formal risk-of-bias assessment was conducted.

**Results:**

A total of 50 studies published between 2012 and 2026 met the eligibility criteria. Eight social media platforms were represented, with X (formerly Twitter; X Corp) most frequently studied, followed by Weibo (Weibo Corporation), and YouTube (Google LLC). HPV vaccine–related communication content was synthesized into 5 categories: risk- and concern-oriented, benefit- and prevention-oriented, misinformation- and distrust-related, experiential and narrative, and practical and policy-related communication. Across studies, prevention and benefit messages coexisted with safety concerns, side effects, access barriers, misinformation, distrust, and personal narratives. Personal accounts were often associated with experiential narratives or negative sentiment, whereas professional sources were less common but generally provided more informative or higher-quality content. Analytical approaches included content analysis, sentiment analysis, topic modeling, and network analysis, but theoretical frameworks were applied in only a minority of studies.

**Conclusions:**

The evidence was limited by heterogeneity in platforms, sampling strategies, data collection periods, coding schemes, and analytical methods, limiting direct comparison across studies. Because most studies analyzed publicly available social media content, this review could not determine individual-level exposure, interpretation, vaccination intention, or vaccine uptake. Overall, HPV vaccine discourse cannot be adequately understood through sentiment polarity alone. This review provides a cross-platform, content-focused synthesis of how HPV vaccine–related social media communication has been studied. Unlike previous reviews, this review clarifies how HPV vaccine–related content has been described, who generates it, and which methods have been used to analyze it. These findings support theory-informed, platform-sensitive research and may inform public health communication strategies, including evidence-based education, transparent risk communication, credible source engagement, narrative-informed messaging, misinformation monitoring, and improved information quality.

## Introduction

### Rationale

Cervical cancer remains one of the most common cancers among females worldwide [1], with an estimated 600,000 new cases and more than 300,000 deaths annually [2]. Persistent infection with high-risk types of human papillomavirus (HPV) is recognized as the primary cause of nearly all cervical cancer cases, with HPV types 16 and 18 accounting for approximately 70% of cases [3]. HPV infection itself is one of the most prevalent sexually transmitted infections globally, with particularly high prevalence among adolescents and young adults, especially soon after sexual debut [4].

Prophylactic HPV vaccines have been developed to prevent infection with high-risk HPV types and are available in bivalent, quadrivalent, and nonavalent formulations, covering different combinations of HPV types, including oncogenic types 16 and 18 and, in some formulations, additional oncogenic or wart-associated types [5]. These vaccines have demonstrated high efficacy in preventing HPV infection and cervical precancerous lesions, particularly when administered before exposure [6]. Importantly, HPV vaccination contributes not only to the prevention of cervical cancer but also to the prevention of other HPV-related cancers, including vaginal, vulvar, penile, anal, and oropharyngeal cancers [7]. The World Health Organization (WHO) recommends routine vaccination for girls aged 9‐14 years as a key strategy for cervical cancer prevention. The WHO has issued a global call to eliminate cervical cancer, with high HPV vaccination coverage as a key pillar of its strategy [8]. However, vaccination efforts face significant challenges, and coverage rates in many countries remain low. Contributing factors include limited public awareness [9-11], economic barriers [12,13], and persistent concerns regarding vaccine safety [9]. These persistent barriers suggest that HPV vaccination is not only a biomedical prevention issue but also a communication challenge, in which public understanding, perceived risk, trust, and access-related information may shape vaccine-related decision-making [9-13].

In recent years, social media platforms, as part of the digital public health ecosystem, have emerged as major sources of health information in daily life. The WHO has recognized digital technologies, including social media, as essential tools for strengthening health service delivery and empowering individuals to make health-enabling decisions. Social media can support human-centered, digitally enabled health systems by improving health literacy and fostering inclusive communication approaches [14]. Platforms such as YouTube (Google LLC), X (formerly Twitter; X Corp), Facebook (Meta Platforms, Inc), and TikTok (ByteDance Ltd) attract vast user bases, especially among younger generations [15]. These platforms enable rapid dissemination, user engagement, and real-time access to information.

However, while social media platforms have the potential to improve health literacy and promote preventive behaviors, they also contribute to the spread of misinformation and can skew toward emotionally charged narratives and anecdotal accounts lacking scientific validity. Such content can significantly influence public attitudes and behaviors [16]. In addition, although multiplatform dissemination may expand audience reach, it can intensify content fragmentation. Compared with single-channel interventions, multiplatform dynamics may undermine information consistency and complicate public health response strategies. Therefore, evaluating the nature and quality of health information shared on social media is critical for developing effective public health communication strategies.

Previous reviews can be grouped into 3 related areas: general assessments of health information quality on social media, reviews of HPV-related social media research, and reviews of the influence of social media on HPV vaccine–related knowledge or attitudes. First, reviews of health information on YouTube have reported generally low information quality and the presence of misinformation [17,18]. Second, a previous review of HPV vaccine–related social media studies published up to 2018 evaluated platform type, sentiment, content accuracy, creator classification, and potential influence on public opinion [19]. Third, one scoping review examined social media research related to HPV, cervical cancer, and screening across multiple platforms, but its primary aim was to map how social media had been used in research rather than to synthesize content characteristics, information sources, or analytical approaches [20]. Finally, a recent systematic review of adolescents and young adults found that social media has mixed effects on HPV vaccine–related knowledge and attitudes, with both beneficial influences and negative impacts linked to misinformation exposure [21].

In parallel with these developments, recent advances in computational approaches, particularly natural language processing (NLP), have expanded how health-related social media content can be analyzed, including misinformation detection, sentiment classification, topic modeling, and information-quality assessment. However, previous reviews have not sufficiently synthesized how these analytical approaches have been applied to HPV vaccine–related social media research across platforms [22]. Overall, existing reviews vary in scope and analytical depth and remain limited by a focus on a single platform, reliance on relatively outdated evidence, or insufficient synthesis of content characteristics.

To address these gaps, this scoping review synthesizes HPV vaccine–related social media research across multiple platforms. Unlike previous reviews, which have primarily focused on single platforms, descriptive analyses, or literature mapping, this study systematically examines communication content, information sources, and analytical approaches, thereby offering an integrated understanding of how HPV vaccine–related information is generated, distributed, and analyzed in the evolving social media environment.

### Objectives

This review aims to systematically map and synthesize existing research on HPV vaccine–related social media content, with particular focus on communication patterns, information sources, and analytical approaches. Specifically, the following research questions (RQs) are addressed:

- RQ1: What types of HPV vaccine–related communication content have been reported across social media platforms?
- RQ2: How do HPV vaccine–related communication patterns differ across social media platforms and information sources, such as individuals, health professionals, and organizations?
- RQ3: How have previous studies applied analytical approaches and theoretical frameworks to examine HPV vaccine communication on social media?

## Methods

### Protocol and Registration

This review adhered to the PRISMA-ScR (Preferred Reporting Items for Systematic Reviews and Meta-Analyses extension for Scoping Reviews) guidelines [23] to ensure methodological rigor and transparency. The completed checklist is provided in Checklist 1. The protocol was prospectively registered with the Open Science Framework [24].

### Eligibility Criteria

The eligibility criteria followed the Population-Concept-Context (PCC) framework in accordance with Joanna Briggs Institute (JBI) guidance [25].

The population or source of evidence comprised HPV vaccine–related social media content, including information, discussions, and user-generated posts addressing HPV vaccination or related topics. The concept of interest was health information and communication about HPV vaccination, including content characteristics (eg, themes, sentiment, misinformation, or information quality) and communication patterns. The context was restricted to social media platforms, including YouTube, X, TikTok, Facebook, Instagram (Meta Platforms, Inc), and other user-generated content platforms. Studies were included if they (1) reported original research, (2) were published in peer-reviewed journals, and (3) were written in English and published between 2006, when HPV vaccination became available, and June 10, 2026.

Studies were excluded if they (1) did not specifically examine HPV vaccine-related content, (2) did not analyze social media–based data, (3) focused solely on methodological or tool development without content analysis, or (4) were nonoriginal publications, such as reviews, editorials, short papers, or conference abstracts.

### Information Sources

Systematic searches were conducted in PubMed, Web of Science, MEDLINE via EBSCOhost, Embase, and Scopus. The final database search was performed on June 10, 2026.

### Search

The search strategy was developed using a combination of controlled vocabulary and free-text terms, in accordance with methodological guidance from the Cochrane Handbook [26] and JBI Manual [25]. Specifically, database-specific controlled vocabulary, including MeSH terms in PubMed, MeSH terms in MEDLINE via EBSCOhost, and Emtree terms in Embase, was combined with free-text keywords related to HPV vaccination and social media. Boolean operators (AND/OR), truncation (*), and database-specific field tags or modifiers, including TS in Web of Science, TITLE-ABS-KEY in Scopus, Title/Abstract in PubMed, TI and AB in MEDLINE, and ti/ab/kw in Embase, were applied. The full database-specific search strategies are provided in Multimedia Appendix 1.

In addition to database searches, backward and forward citation searching was performed for all included studies after full-text screening. Backward citation searching was conducted by screening the reference lists of the included studies, using reference lists available in Scopus and, where necessary, the full texts of the included studies. Forward citation searching was conducted using Scopus to identify records that cited the included studies. Records identified through citation searching were exported, deduplicated, and screened using the same eligibility criteria as the database search. Records already identified through database searches were removed before screening. Citation searching was completed on July 17, 2026.

The search process was reported in accordance with PRISMA-S (Preferred Reporting Items for Systematic Reviews and Meta-Analyses literature search extension) [27] to strengthen transparency. The PRISMA-S checklist is provided in Checklist 2.

### Selection of Sources of Evidence

Two independent reviewers screened titles and abstracts of papers identified through the database searches and citation searches according to the inclusion and exclusion criteria. Discrepancies between the 2 reviewers were resolved through discussion, and the full text of the paper was consulted when abstracts lacked sufficient information. Full-text articles were assessed for eligibility by one reviewer, and the eligibility decisions were verified by a second reviewer. Any disagreements were resolved through discussion and consensus.

### Data Charting Process

Data charting was performed using a structured extraction form developed in accordance with JBI guidance [25]. The extraction form was reviewed and refined during the charting process to ensure consistent capture of methodological components and findings relevant to the review questions across studies. One reviewer charted data from all included studies, and a second reviewer verified the completed entries against the full-text articles to ensure completeness and accuracy. Any discrepancies or uncertainties were resolved through discussion and re-examination of the original articles.

### Data Items

The extraction form captured key study characteristics and variables relevant to the review questions, including (1) publication details (title, authors, publication year, DOI, and journal); (2) platforms analyzed; (3) purpose and aim; (4) search keywords; (5) content languages; (6) number of content items analyzed; (7) data period and data collection date or period; (8) analytical methods, including the primary analytical method and additional methodological components; (9) main findings related to HPV vaccine communication; (10) study-reported themes or content findings; and (11) theoretical frameworks. Data were recorded and managed using Microsoft Excel (Microsoft Corp). The data-charting form is provided in Multimedia Appendix 2.

### Critical Appraisal of Individual Sources of Evidence

Consistent with scoping review methodology, this study did not conduct a formal critical appraisal of the methodological quality or risk of bias of individual sources of evidence. The aim was to map the extent, range, and nature of existing research on HPV vaccine–related social media content, rather than to assess intervention effectiveness or generate pooled effect estimates. Therefore, no studies were excluded on the basis of methodological quality.

### Synthesis of Results

Extracted data were synthesized descriptively in accordance with scoping review methodology. Frequency counts were used to summarize study characteristics, including publication year, social media platform, content language, analytical approach, information source, and use of theoretical frameworks. Study-level characteristics and main findings were tabulated for each included study.

To synthesize content-related findings, the themes, categories, and communication patterns reported in the included studies were compared and grouped according to conceptual similarity. The synthesis was based on author-reported findings rather than a reanalysis of the original social media content. Categories were not mutually exclusive, and each study was counted once in every applicable category. Sentiment, information-source, information-quality, and engagement findings were summarized according to the classifications and assessment approaches reported in the original studies. Reported differences across platforms and information-source types were summarized narratively.

Visualizations were developed to display publication trends, platform distribution, and analytical approaches across platforms. Studies examining multiple platforms were counted once for each applicable platform in platform-based visualizations. For studies using multiple analytical components, the primary method used to generate the main findings was selected for the gap map, and approaches were classified as manual or automated.

## Results

### Selection of Sources of Evidence

**Figure 1.**
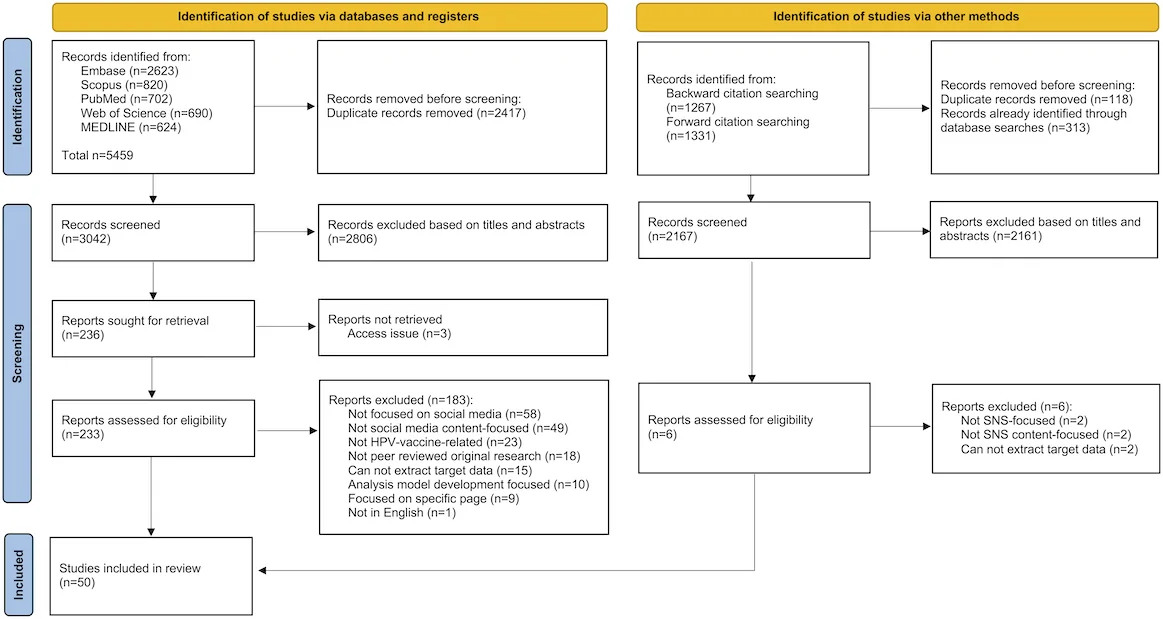
PRISMA (Preferred Reporting Items for Systematic Reviews and Meta-Analyses) 2020 flow diagram of the study selection process. HPV: human papillomavirus; SNS: social networking service.

A total of 5459 records were retrieved from the database search. After removing 2417 duplicates based on study titles and DOIs, 3042 records underwent title and abstract screening. During title and abstract screening, 2806 records were excluded for the following reasons: 1117 were not peer-reviewed original articles, 793 were unrelated to social media platforms, 694 were unrelated to HPV vaccination, and 202 did not examine communication content. After 3 reports were excluded because full texts were unavailable, 233 papers were reviewed in full text, and 50 met the eligibility criteria and were included in the final review. Backward citation searching identified 1267 records from the reference lists of included studies, and forward citation searching identified 1331 citing records in Scopus. After removing 118 duplicate records and 313 records already identified through database searches, 2167 records were screened. Of these, 2161 records were excluded during title and abstract screening, and 6 reports were assessed for eligibility. All 6 reports were excluded because they were not focused on social media (n=2), did not focus on social media content (n=2), or did not allow extraction of target data (n=2). No additional eligible studies were identified through citation searching. Figure 1 illustrates the study selection process. The diagram summarizes the identification, screening, eligibility assessment, and inclusion of peer-reviewed original studies on HPV vaccine-related social media content in this scoping review. Searches were conducted in PubMed, Web of Science, MEDLINE, Embase, and Scopus. Backward and forward citation searching was also conducted, but no additional eligible studies were identified. The eligibility period covered studies published between 2006 and 2026, and 50 studies published between 2012 and 2026 [28-77] were included in the final synthesis.

### Characteristics of Sources of Evidence

**Figure 2.**
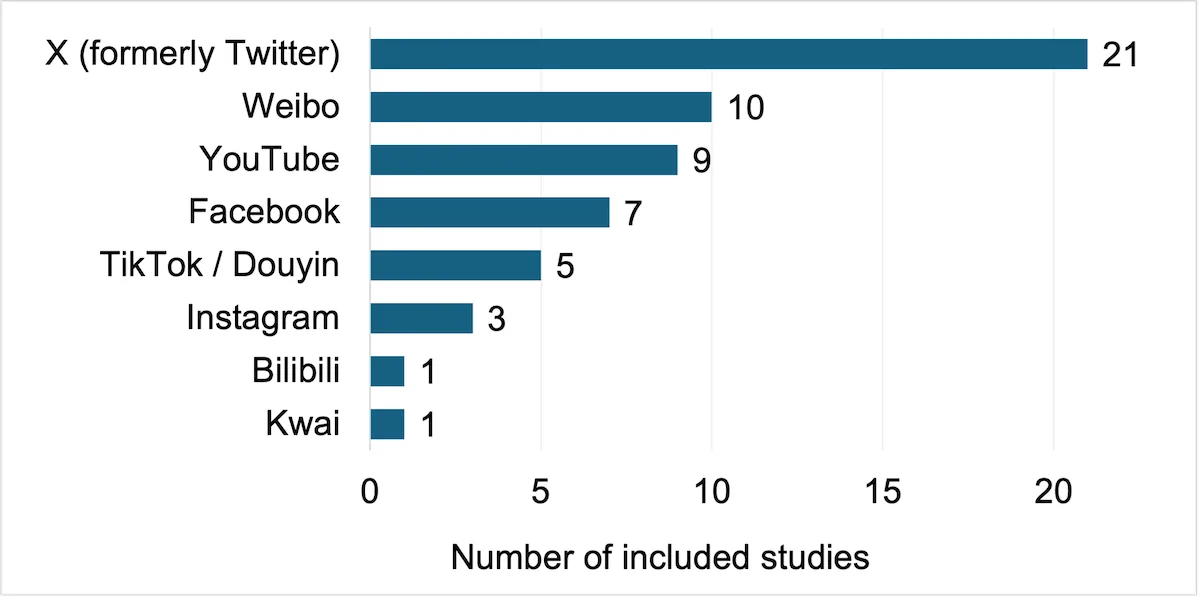
Distribution of social media platforms analyzed in included studies.

The characteristics of the included studies are summarized in Table 1. The earliest study, published in 2012, analyzed 172 English-language YouTube videos [28]. Eight social media platforms were analyzed across the included studies, with X the most frequently examined (n=21) [29-36,38,40,41,43,48,55,58,59,63-65,71,72], followed by Weibo (Weibo Corporation; n=10) [44,46,49,51,54,56,67,69,74,76] and YouTube (n=9) [28,37,57,62,68,72,73,75,77]. Figure 2 illustrates the distribution of studies across platforms, highlighting the dominance of X and the limited representation of emerging platforms such as Bilibili (Bilibili Inc) and Kwai (Kuaishou Technology). The bar chart shows the number of included studies analyzing HPV vaccine–related communication content on each platform. Studies that analyzed more than one platform were counted once for each relevant platform; therefore, the total platform count exceeds the number of included studies.

**Table 1.** Characteristics and main findings of included studies on HPV^a^ vaccine–related social media content^b^.

Study	Social media platforms	Content language	Analyzed content sample size	Main findings relevant to HPV vaccine communication
Briones et al (2012) [28]	YouTube	English	172	Most HPV vaccine–related YouTube videos were predominantly negative (defined as content that disapproved of or opposed HPV vaccination) and frequently invoked conspiracy and civil-liberty themes, while receiving greater viewer approval than positive content.
Dunn et al (2015) [29]	Twitter	English	83,551	Exposure to predominantly negative HPV vaccine opinions within Twitter communities was associated with a substantially higher likelihood of users subsequently expressing negative opinions (RR^c^=3.46, 95% CI 3.25‐3.67, *P*<.001).
Surian et al (2016) [30]	Twitter	English	284,303	HPV vaccine–related opinions on Twitter were clustered into distinct communities, with some communities showing greater vulnerability to negative influence.
Massey et al (2016) [31]	Twitter	English	193,379	HPV vaccine–related discussions on Twitter were predominantly positive, with positive and neutral tweets focused more on prevention, while negative tweets disproportionately emphasized side effects.
Du et al (2017) [32]	Twitter	English	184,214	Public sentiment toward HPV vaccines on Twitter showed temporal fluctuations, with negative sentiment initially decreasing and later increasing, while concerns about vaccine efficacy declined over time.
Shapiro et al (2017) [33]	Twitter	English	43,852	The proportion and types of HPV vaccine concerns expressed on Twitter were similar across Australia, Canada, and the United Kingdom, with users expressing concerns more strongly connected to international users sharing similar views.
Keim-Malpass et al (2017) [34]	Twitter	English	1794	HPV vaccine–related tweets were highly polarized, with lay consumers and political or religious users more likely to post negative content, while health advocates and news media more often posted positive content.
Chakraborty et al (2017) [35]	Twitter	English	1887	HPV vaccine–related tweets frequently focused on news or media coverage, sexual behavior, vaccine safety, and vaccine effectiveness, with misinformation such as gender-specific vaccine myths also observed.
Massey et al (2018) [36]	Twitter	English	193,379	Health professionals’ HPV vaccine–related tweet spikes were driven by newly published scientific evidence, whereas parent-directed tweet spikes were driven by awareness events and more often included personal experiences or opinions.
Ekram et al (2019) [37]	YouTube	English	35	HPV vaccine–related YouTube videos were predominantly negative, with antivaccine videos more likely to contain inaccurate or incomplete information, and comments frequently emphasized side effects and conspiracy theories.
Luo et al (2019) [38]	Twitter	English	287,100	Public opinion on HPV vaccination on Twitter fluctuated over time, with periods of heightened negativity and recurring negative themes centered on safety concerns, adverse effects, and scandals.
Kearney et al (2019) [39]	Instagram	English	360	Although provaccine content was more prevalent on Instagram, antivaccine posts, which often featured personal narratives and misleading information, received substantially higher user engagement.
Zhang et al (2020) [40]	Twitter	English	2,598,033	Twitter discussions about HPV vaccination reflected 6 constructs of the integrated behavior model: feelings about behavior; behavioral beliefs; normative beliefs regarding others’ behavior; knowledge; normative beliefs regarding others’ expectations; and environmental constraints. Several topic distributions were significantly correlated with the Health Information National Trends Survey responses, suggesting that Twitter data can capture HPV vaccine-related public attitudes at scale.
Himelboim et al (2020) [41]	Twitter	English	39,368 users	HPV vaccine discussions on Twitter formed distinct user clusters with different emotional and content characteristics: provaccine clusters were characterized by positive emotion and institutional sources, whereas antivaccine clusters showed more negative emotion and questionable sources. Positive emotion was associated with denser information sharing, while anger weakened cluster cohesion.
Luisi (2020) [42]	Facebook	English	6506	HPV vaccine–related Facebook posts were predominantly negative, focused more on perceived barriers than benefits, and antivaccine content consistently generated the highest engagement.
Du et al (2020) [43]	Twitter	English	1,431,463	Public perceptions of the HPV vaccine on Twitter shifted over time toward fewer perceived barriers and more positive attitudes, with substantial geographic variation and persistently higher negative perceptions in certain US states.
Chen et al (2020) [44]	Weibo	Chinese	1894	Media discourse on HPV vaccination in China was generally positive but misleading, with inconsistent health belief messaging and emerging conspiracy narratives rooted in nationalism.
Massey et al (2020) [45]	Instagram	English	580	On Instagram, anti-HPV vaccine posts were more likely to originate from individuals and include personal narratives, with misinformation themes including concealment, conspiracy theories, unsubstantiated claims, vaccine inefficacy, and vaccine-related injury; provaccine posts were more often organizational and informational.
Wang et al (2020) [46]	Weibo	Chinese	67,569	Most posts were positive or neutral, while negative posts revealed that Chinese women were generally willing to receive HPV vaccination, but significant geographic disparities existed. Key concerns centered on access barriers, policy restrictions, and vaccination logistics.
Zhang et al (2021) [47]	Facebook	English	212	HPV vaccine promotion on Facebook was limited in frequency and content diversity. Posts mainly focused on awareness, screening, information sources, and calls to action, whereas comments more often discussed vaccine hesitancy, safety concerns, vaccine risks and injuries, distrust in vaccine science, and misinformation.
Suzuki et al (2021) [48]	Twitter	Japanese	208	HPV vaccine–related information on Twitter in Japan was disseminated by a small number of users and frequently lacked verifiable accuracy; many posts contained personal opinions rather than reliable medical information.
Zhou et al (2021) [49]	Weibo	Chinese	1164	HPV vaccine discussions on Weibo in China were dominated by low-influence, lay-user content that emphasized vaccine type differences, such as price and availability, while giving little attention to safety and effectiveness.
Luisi (2021) [50]	Facebook	English	6506	HPV vaccine content on Facebook is dominated by negatively toned, user-generated posts that amplify perceived risk, are more likely to include hyperlinks, receive higher engagement, and exhibit temporal momentum, indicating a self-reinforcing pattern of risk amplification on social media.
Jiang et al (2021) [51]	Weibo	Chinese	67,773	HPV vaccine content on Chinese social media Weibo is dominated by advertising, guidance information, and personal experience sharing, with smaller proportions of policy, scandal, and risk-related content, and engagement with these information types through scanning, seeking, and discussion is significantly associated with users’ knowledge, attitudes, and intention to vaccinate.
Sobeczek et al (2022) [52]	Facebook	Polish	1442	Polish-language HPV vaccination discourse on Facebook was dominated by centralized negative sentiment driven by influential antivaccine leaders, with no clear change in overall interest during the COVID-19 pandemic.
Boatman et al (2022) [53]	TikTok	English	170	Most HPV vaccine–related TikTok videos expressed provaccine opinions and primarily focused on cancer prevention. Antivaccine videos primarily focused on side effects and generated higher user engagement, while health professionals mainly created educational provaccine content.
Li et al (2022) [54]	Weibo	Chinese	1221	HPV vaccine information on Weibo was inadequately and unevenly represented, dominated by non-expert sources, overemphasized vaccine efficacy, and reinforced gender imbalance by placing prevention responsibility mainly on women.
Boucher et al (2023) [55]	Twitter	English	596,987	HPV vaccine discussions on Twitter remained thematically stable during the COVID-19 pandemic; however, negative sentiment intensified within vaccine-hesitant networks while attention to HPV vaccination declined among vaccine-confident groups.
Hu et al (2023) [56]	Weibo	Chinese	6917	HPV vaccine discussions on Weibo reflected diverse user-driven narratives, showing improved public awareness but persistently inadequate public knowledge about HPV.
Di Spirito et al (2023) [57]	YouTube	English	97	Most YouTube videos on HPV-related oral health provided moderately accurate, educational content and predominantly encouraged HPV vaccination, indicating their suitability for mass health communication.
Terada et al (2023) [58]	Twitter	Japanese	3623	Following Japan’s resumption of proactive HPV vaccine recommendations, Twitter discussions were dominated by safety and side-effect concerns with largely negative sentiment, whereas many health professionals posted neutral rather than explicitly supportive messages.
Kornides et al (2023) [59]	Twitter	English	3876	About one-quarter of HPV-related tweets contained misinformation, primarily about adverse health effects, mandatory vaccination, and vaccine inefficacy, and these posts, especially those using personal narratives or referencing children, received substantially higher engagement and were significantly more likely to be retweeted than supportive content.
McKenzie et al (2024) [60]	Facebook	English	138	Highly engaged HPV vaccine articles on Facebook showed distinct persuasive patterns. Provaccine articles mainly used evidence on vaccine benefits and cancer prevention, whereas antivaccine articles were concentrated among a small number of sources and used fear appeals, mistrust of institutions, ideological appeals, and claims minimizing the severity of HPV.
Lin et al (2024) [61]	TikTok	English	131	HPV vaccination content on TikTok was generally low in informational quality, even when physicians were involved, and frequently omitted key messages about eligibility, male vaccination, and non-cervical cancer prevention.
Xu et al (2024) [62]	YouTube	Japanese	334	Japanese YouTube content on HPV vaccines showed a shift from predominantly negative attitudes around 2013 to mostly positive attitudes after 2019; negative videos were mainly produced by individuals, whereas professional sources posted only positive or neutral content.
Boatman et al (2024) [63]	Twitter, Facebook, and TikTok	English	Twitter 998, Facebook 999, TikTok 999	HPV vaccine misinformation was widespread in social media comment sections, with common themes such as adverse reactions and conspiracy theories varying in prevalence across platforms.
Rai et al (2024) [64]	Twitter	English	Approximately 653,000	HPV vaccine discourse on social media is dominated by concerns about adverse effects, personal experiences, and vaccine mandates, with a notable post-2020 shift toward more personalized narratives, especially parental concerns and anecdotal reports of vaccine injury, indicating an increasingly individualized and evolving pattern of vaccine hesitancy.
Rajkhowa et al (2024) [65]	Twitter	English	1487	HPV vaccine discourse on social media in India shows mixed but generally positive sentiment, alongside widespread misinformation and concerns, particularly about safety, efficacy, ethical issues, and accessibility, reflecting significant uncertainty and information gaps that may contribute to vaccine hesitancy.
Su et al (2025) [66]	Kwai, Bilibili, and TikTok	Chinese	238	The quality and reliability of HPV vaccine-related videos varied across Chinese short-video platforms. TikTok videos had the highest GQS and mDISCERN scores and the highest median likes and shares, while videos uploaded by professionals showed higher quality, greater reliability, and more positive public responses than videos uploaded by individual users.
Shi et al (2025) [67]	Weibo	Chinese	38,615	Public emotions toward HPV vaccination on Weibo showed increasing polarization over time, with skepticism about commercialization amplifying anger, whereas university-led outreach fostered positive emotions through more centralized communication networks.
Özeller et al (2025) [68]	YouTube	Turkish	81	Turkish-language YouTube videos on HPV vaccination, although largely produced by healthcare professionals, showed only moderate quality and limited effectiveness in promoting vaccination.
Zhou et al (2025) [69]	Weibo	Chinese	4997	Weibo discussions on male HPV vaccination center on 5 frames: disease risk/prevention, virus transmission, gender roles, vaccine promotion/acceptance, and market consumption. Although awareness and acceptance of male HPV vaccination appeared to be increasing, gendered misconceptions, limited scientific understanding, high costs, commercial distrust, and delayed policy approval continue to hinder equitable male HPV vaccination in China.
Matthews et al (2025) [70]	TikTok	English	200	HPV vaccine–related TikTok videos were largely provaccination and often created by health care professionals. However, many videos lacked comprehensive and accurate educational information, especially regarding noncervical cancers, vaccine safety, and eligibility, despite high engagement.
Liu et al (2025) [71]	Twitter	Japanese	228,300	HPV vaccine discourse on Japanese social media shows event-driven fluctuations in public attitudes. Safety concerns peaked around major controversies, discussions of vaccine effectiveness increased over time, and misinformation remained present but relatively low. Attitudes toward HPV and COVID-19 vaccines were also interconnected, suggesting dynamic and context-dependent patterns of vaccine hesitancy.
Huang et al (2025) [72]	YouTube, Facebook, Twitter/X, Instagram	English	56	Pro-HPV vaccination videos differed by video type, with animation videos emphasizing vividness and misinformation rebuttal, produced-story videos emphasizing disease severity, and velfie, videos showing higher believability and authenticity.
Yanmaz and Gursoy (2026) [73]	YouTube	Turkish	26	Physicians emphasized the safety, effectiveness, and importance of HPV vaccination while correcting common misconceptions. Limited access due to the vaccine’s exclusion from the national immunization program was identified as a major barrier.
Zhang et al (2026) [74]	Weibo	Chinese	3,53,530	Five themes of HPV vaccine discussions were identified. Following the implementation of 2 national HPV vaccination policies, discussions on vaccine accessibility decreased, whereas discussions on HPV awareness, knowledge, and gender-related issues increased.
Yuzugullu and Kiziltas (2026) [75]	YouTube	English	113	Most videos supported HPV vaccination. Videos produced by health care professionals or organizations had significantly higher quality and reliability scores than those produced by nonexpert or media sources, and longer videos tended to have higher quality and reliability scores.
Ding et al (2026) [76]	Weibo	Chinese	817,788	Practical barriers were discussed more frequently than perceived barriers. The most common barriers included limited 9-valent vaccine supply, age restrictions, long waiting times, and concerns about vaccine adverse effects.
Gülmez and Ciftci (2026) [77]	YouTube	Turkish	270	More than half of the videos were produced by health care professionals and most recommended HPV vaccination. Videos produced by health care professionals and longer videos had higher information quality and reliability, whereas video popularity was not associated with information quality.

a HPV: human papillomavirus.

b The table summarizes the 50 studies included in this scoping review, which were published between 2012 and 2026 and analyzed social media content across 8 platforms. Each study is summarized by platform, content language, sample size, and content type, and main findings related to communication content, sentiment, information sources, misinformation, or information quality.

c RR: relative risk.

Of the 50 studies [28-77], 31 analyzed English-language content [28-43,45,47,50,53,55,57,59-61,63-65,70,72,75], 11 analyzed Chinese-language content [44,46,49,51,54,56,66,67,69], 4 focused on Japanese-language content [48,58,62,71], 3 analyzed Turkish-language content [68,73,77], and 1 analyzed Polish-language content [52]. Overall, most studies examined a single social media platform, providing descriptive analyses of the publicly available content.

### Critical Appraisal Within Sources of Evidence

Because no formal critical appraisal was conducted for this scoping review, no critical appraisal results are presented.

### Results of Individual Sources of Evidence

The study-level characteristics and main findings of each included source of evidence are presented in Table 1.

**Figure 3.**
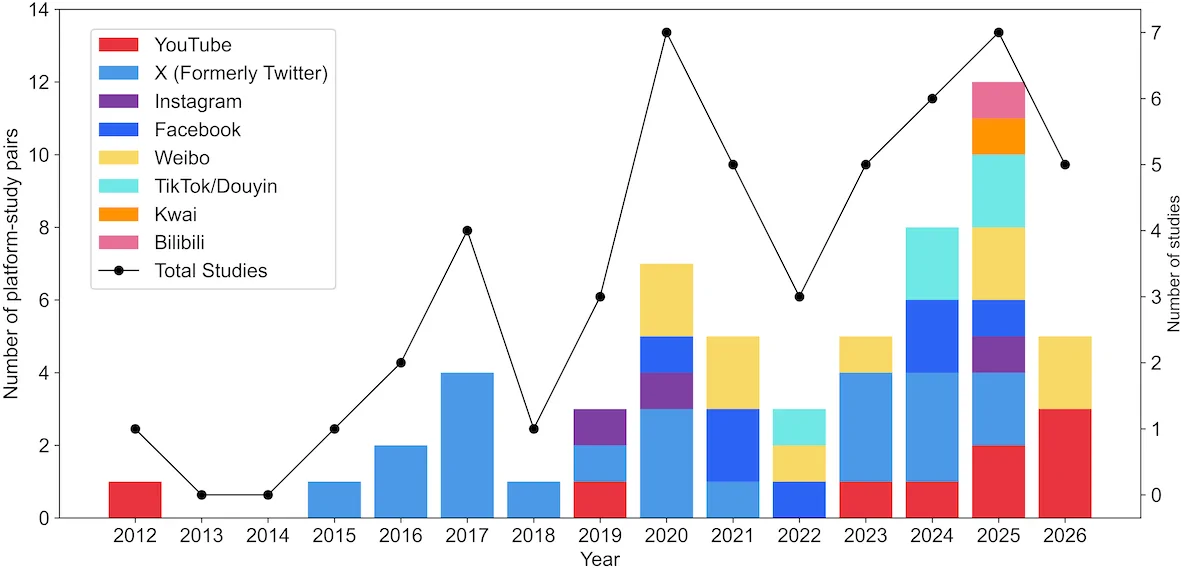
Annual publication trends and platform distribution of included studies. No eligible studies published between 2006 and 2011 were identified. Data for 2026 include only studies published up to June 10, 2026.

Figure 3 illustrates the annual publication trend from 2012 to 2026 and the distribution of social media platforms studied. Stacked bars represent platform-study pairs, with studies analyzing multiple platforms counted once for each relevant platform. The black line represents the total number of included articles published each year. The *x*-axis begins in 2012 because no eligible studies were identified between 2006 and 2011. The total number of platform-study pairs was counted, with each platform analyzed within a study counted separately. The number of studies published each year increased from 2015, with a marked and sustained rise after 2019, despite year-to-year fluctuations. Studies published after 2019 also expanded in scope to include Instagram, Facebook, Weibo, TikTok, and later Kwai and Bilibili.

### Synthesis of Results

#### Communication Content Related to the HPV Vaccine (RQ1)

Across the included studies, HPV vaccine–related social media content was organized into five broad communication categories: (1) risk- and concern-oriented communication, (2) benefit- and prevention-oriented communication, (3) misinformation- and distrust-related communication, (4) experiential and narrative communication, and (5) practical and policy-related communication. These categories were not mutually exclusive; studies could report more than one communication category. Despite differences in platforms, coding schemes, and analytical approaches, these categories reflected recurring patterns in how HPV vaccination was discussed across social media platforms. These 5 communication categories are summarized in Table 2.

**Table 2.** Synthesized categories of communication content reported in the included studies^a^.

Category	Studies, n	Platforms represented	Examples of content
Risk- and concern-oriented communication	38	X, Facebook, Weibo, YouTube, TikTok, and Instagram	Safety concerns, side effects, perceived risks, doubts about effectiveness, and concerns related to sexual behavior and sexuality
Benefit- and prevention-oriented communication	34	X, Weibo, TikTok, Facebook, YouTube, and Instagram	Cancer and other HPV^b^-related disease prevention, vaccine effectiveness, vaccination promotion, HPV and vaccine awareness, and male vaccination
Experiential and narrative communication	29	X, Weibo, Instagram, YouTube, TikTok, and Facebook	Personal experiences, user stories, parental concerns, and vaccine decision-making narratives
Misinformation- and distrust-related communication	23	X, YouTube, Facebook, Weibo, Instagram, and TikTok	Conspiracy theories, mistrust, inaccurate or misleading information, distrust of authorities or commercialization, and misinformation rebuttal
Practical and policy-related communication	23	Weibo, X, TikTok, Facebook, YouTube	Availability and access, cost and appointment barriers, age and gender eligibility, vaccine type, insurance or subsidy policies, and policy changes

a The table summarizes recurring communication categories identified through descriptive synthesis of the included studies. “n” indicates the number of studies reporting each category. Because individual studies could report multiple communication categories, categories were not mutually exclusive, and each study was counted in every applicable category.

b HPV: human papillomavirus.

Risk- and concern-oriented communication was most frequently reported (n=38). This category comprised safety concerns, side effects, adverse reactions, perceived risks and barriers, fear of long-term consequences, doubts about vaccine effectiveness, sexual behavior–related concerns, access-related concerns, and vaccine hesitancy. These patterns appeared across studies of X, Facebook, Weibo, YouTube, TikTok, and Instagram [28,30-38,40,42,44-47,50-53,55,56,58-60,62-67,69-74,76].

Benefit- and prevention-oriented communication was also frequently reported (n=34). This content framed HPV vaccination as a means of disease prevention, including cervical cancer prevention, prevention of other HPV-related diseases, vaccine effectiveness, vaccine promotion, male vaccination, and vaccination acceptance. These themes appeared across studies of X, Weibo, TikTok, Facebook, YouTube, and Instagram [28,31,34-36,38,40,42,44,46,47,49,51-58,60-62,64-66,68-74,77].

Experiential and narrative communication was another recurring category (n=29). This category included personal experiences, user stories, parental concerns, and experience-based accounts of vaccination decision-making. Such narratives appeared in both supportive and hesitant or antivaccine contexts, although several studies reported that antivaccine, or misinformation-related narratives were associated with higher engagement [28,30,34-37,39,40,45,46,48,49,51,53,55,56,58,59,62-65,67-70,72,74,77].

Misinformation- and distrust-related communication was also prominent (n=23). This category comprised conspiracy theories, mistrust, misleading information, distrust of authorities or commercialization, doubts about vaccine effectiveness, ideological appeals, and corrective communication addressing misinformation. These patterns were reported across X, YouTube, Facebook, Weibo, Instagram, and TikTok [28,30,34,35,37,39,44,45,47,50,51,55,59,60,63,65,67,68,71-74,76]. Eight studies further indicated that misinformation or antivaccine content was often embedded in personal narratives and was associated with higher user engagement than supportive or neutral content [37,39,45,50,53,59,60,63].

Twenty-three studies also identified practical and policy-related communication, including vaccine access, cost, appointment availability, vaccine type, eligibility, age and gender restrictions or recommendations, vaccine supply, and policy changes. These issues were reported in contexts where vaccine availability, affordability, or policy changes shaped public discussion [33,35,36,46,47,49,51,52,54-56,58,61,64,65,67,69-74,76]. These findings suggest that HPV vaccine–related discussions reflected not only attitudes toward vaccination but also structural and logistical barriers.

In addition to these thematic communication categories, 28 studies [28,29,31,32,34-39,41-44,46,47,50,52,55,58,60,62,65-67,70,71,77] assessed HPV vaccine–related communication using sentiment analysis, defined as identifying the polarity or evaluative orientation expressed in text, commonly as positive, neutral, or negative [78]. Sentiment patterns varied across platforms and contexts: 11 studies reported predominantly positive or provaccine content [31,34,36,39,44,46,62,65,66,70,77], whereas 10 studies identified predominantly negative content or used concern-based classifications that grouped posts by specific concerns, such as safety, side effects, effectiveness, distrust, or access barriers, rather than by positive or negative tone alone [28,35,37,38,42,47,50,52,55,58]. The remaining 7 studies described mixed, fluctuating, or polarized sentiment patterns [29,32,41,43,60,67,71]. Several studies further showed that sentiment changed over time [32,38,43,50,55,62,67,71], differed across communities or network clusters [29,41,52,55,58], or varied by geographic context [43,46,65].

A smaller number of studies assessed information quality, accuracy, or completeness, using established evaluation scales such as the Video Information and Quality Index [57,68], Global Quality Score [57,66,68,70,75], DISCERN [61,63,66,75,77,79], JAMA (*Journal of the American Medical Association*) [57,68,75,77], or a study-specific standard [72]. These studies generally found that HPV vaccine–related social media content was often incomplete or of low to moderate quality, particularly regarding eligibility, male vaccination, vaccine safety, and noncervical HPV-related cancers.

Overall, the reviewed studies indicate that HPV vaccine–related social media communication extended beyond simple positive or negative attitudes. Rather, it comprised overlapping content categories in which prevention benefits, safety concerns, personal narratives, practical barriers, and misinformation coexisted across platforms.

#### Platform- and Source-Related Differences in HPV Vaccine Communications (RQ2)

As summarized in Figure 2, X was the most frequently examined platform. Beyond this distributional pattern, platform-related differences were also evident in analytical focus. As shown in Table 1 and Figure 2, studies of X more often examined sentiment, temporal trends, geographic differences, and network-level communication patterns. In contrast, studies of video- or image-based platforms, such as YouTube, TikTok, Instagram, Bilibili, and Kwai, more often used manual content analysis to assess content characteristics, uploader type, information quality, and engagement. Weibo studies also included computational analyses but tended to emphasize context-specific themes in China, such as access, policy, commercialization, and public concerns.

A total of 22 studies categorized content according to the information source or creator characteristics, such as individuals, health professionals, government agencies, media sources, or other institutional and nonexpert sources [28,34,36,39,41,45,47-50,53,54,58,60-62,66,68,70,72,75,77]. Across these studies, personal or individual accounts were frequently identified as prominent sources of HPV vaccine–related content, followed, depending on the platform, by media sources, organizations, health professionals, and government-affiliated accounts. Four studies reported limited or absent content generated by health professionals or government agencies [39,49,54,62], suggesting that authoritative voices were underrepresented in several social media contexts. Furthermore, 8 studies reported that content from personal or individual accounts was more likely to be associated with negative sentiments, misinformation or experience-based narratives [28,34,39,45,50,53,62,67]. In contrast, content generated by mass media, health professionals, or institutions tended to be positive or generally of higher information quality [34,35,62,66,75,77]. Several studies indicated that source patterns varied by platform: YouTube and X studies more often identified personal or individual accounts as content sources, whereas Weibo studies more often identified mass media or organizational sources.

#### Theoretical Frameworks and Analytical Approaches (RQ3)

Most included studies were descriptive, and theoretical frameworks were applied in only a limited number of studies to guide coding or categorize content. These frameworks included the health belief model (HBM) [28,33,42-45,56,72], information manipulation theory (IMT) [45,63], theory of planned behavior (TPB) [43], elaboration likelihood model (ELM) [60,72], and extended parallel process model (EPPM) [54].

Regarding analytical approaches, nearly all studies used quantitative analysis, often through manual coding of sentiment, content categories, uploader characteristics, information quality, or engagement metrics. Thirty studies used fully manual coding (performed by 2‐4 assessors) for sentiment analysis, content analysis, or information-quality assessment [28,34,35,37,39,42-45,48-50,53,54,56-63,65,66,68,70,72,73,75,77]. Other studies adopted existing artificial intelligence tools or machine learning methods to perform topic modeling, network analysis, and sentiment analysis on a larger scale. Latent Dirichlet allocation (LDA) and the Dirichlet multinomial mixture (DMM) were used for inferring topics, whereas Bidirectional Encoder Representations from Transformers (BERT), Linguistic Inquiry and Word Count (LIWC), and Google Cloud sentiment analysis were used for sentiment analysis.

**Figure 4.**
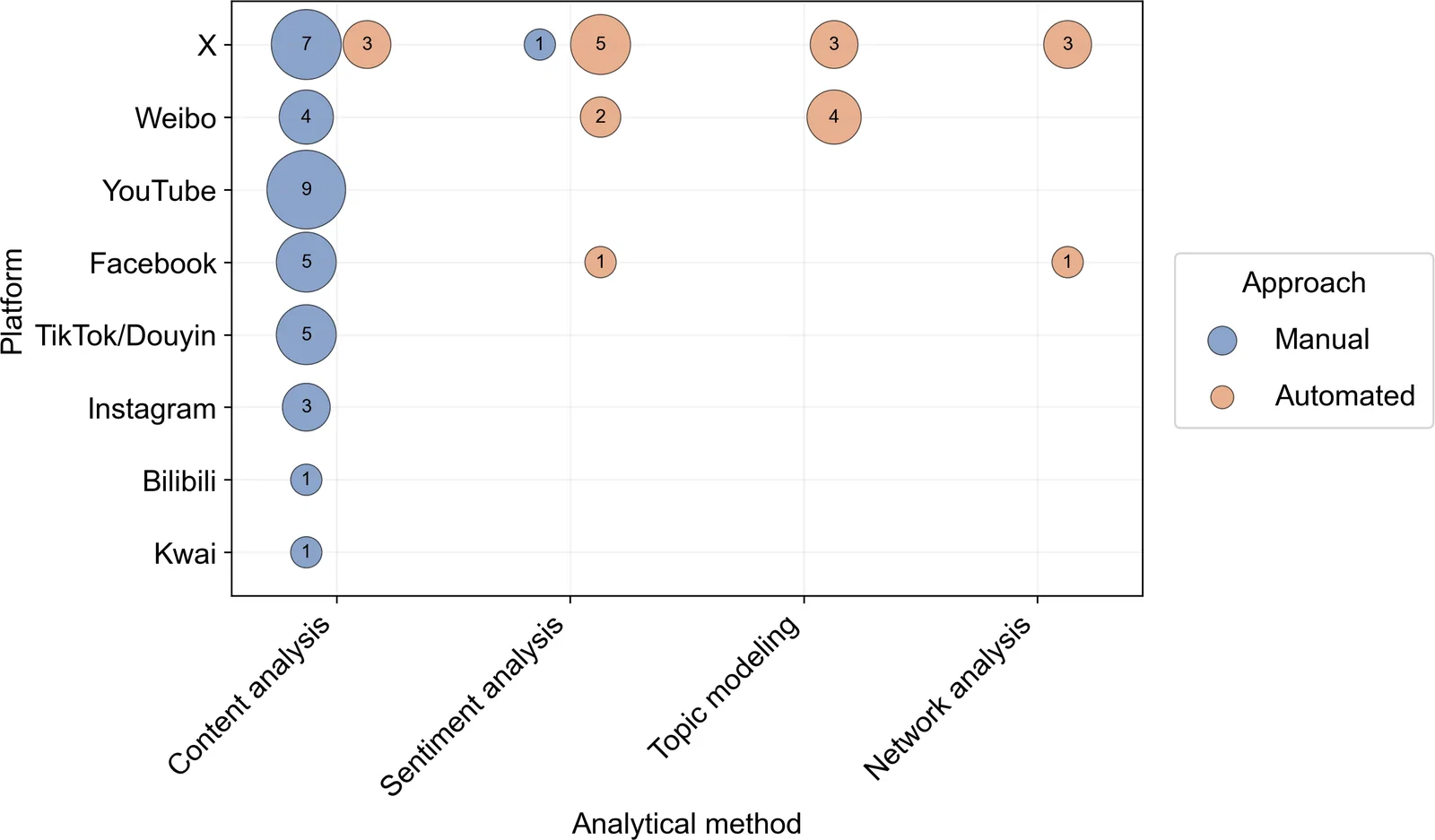
Gap map of analytical methods and approaches across social media platforms.

To visualize methodological patterns across platforms, a gap map was developed to display the distribution of primary analytical methods by social media platform. In Figure 4, the *x*-axis represents the primary analytical method, and the *y*-axis represents the social media platform. Bubble size indicates the number of studies, while bubble color distinguishes manual from automated analytical approaches. Studies using multiple methodological components were classified according to the primary method that generated the core analytical variables.

As shown in Figure 4, manual content analysis was the most frequently used approach and was especially common for video- or image-based platforms, including YouTube, TikTok, Instagram, Bilibili, and Kwai. Automated approaches were concentrated on text-based platforms, such as X and Weibo. X showed the greatest methodological diversity, whereas several newer or less-studied platforms were examined only through manual content analysis. Overall, computational and network-based approaches were concentrated mainly on text-based platforms, whereas manual content analysis predominated in studies of video- and image-based platforms.

## Discussion

### Summary of Evidence

This scoping review mapped HPV vaccine–related social media research across multiple platforms, focusing on communication content, information sources, and analytical approaches. Corresponding to the review questions, the evidence indicates that HPV vaccine–related communication cannot be adequately understood through sentiment polarity alone; rather, it is shaped by the interaction of message content, platform characteristics, information sources, experiential narratives, practical barriers, misinformation, and information quality [28-77]. Although this field has expanded across platforms and methods, the included studies remained largely descriptive, sentiment-focused, and insufficiently theory-informed [19,20,28-77]. These findings suggest that future research may benefit from platform-sensitive and source-aware approaches to studying HPV vaccine communication on social media, given the platform- and source-related differences observed in the included studies [28-77]. They also align with previous reviews showing that social media research on HPV-related topics varies by platform and that social media exposure or interventions may have mixed effects on HPV vaccine–related knowledge, attitudes, and intentions [19-21].

### Interpretation of Content Patterns in HPV Vaccine Communications

The included studies commonly used sentiment analysis, which is consistent with an earlier review [19]. However, this review extends previous work by showing that sentiment polarity alone does not sufficiently capture the range of HPV vaccine–related communication on social media. In addition to sentiment analysis, thematic findings provided further insights into the framing of HPV vaccine–related social media content. Across platforms, themes related to vaccine benefits, cancer prevention, and vaccine effectiveness were consistently reported [31,34-36,38,40,42,46,47,49,53-57,60,61,66,68-70,72-75,77], aligning with dominant health-messaging narratives and determinants of vaccine decisions. However, themes regarding vaccine safety, side effects, perceived risks, access barriers, and distrust were also frequently mentioned [28-39,42,44-47,50-53,55,58-60,62-65,67,69-71,73,74,76], consistent with a previous review [19]. These patterns show that risk-focused concerns persisted alongside benefit-oriented messages, which may help explain why recent reviews reported mixed effects of social media on HPV vaccine–related knowledge, attitudes, and intentions [21]. Taken together, these findings suggest that benefit-oriented and concern-oriented communication should be interpreted as concurrent dimensions of HPV vaccine discourse rather than as mutually exclusive categories.

Several studies also highlighted the prevalence of personal and experiential narratives [37,39,45,50,53,59,60,63]. This finding aligns with previous reviews of YouTube health information, which reported that health-related videos often contain anecdotal or misleading information and that content from government organizations or professional associations tends to be more trustworthy and higher in quality [17,18]. An important feature of social media platforms that facilitates social support [80], such narratives enhance audience engagement and resonance [81]. However, they can also heighten anxiety about risks, particularly when negative personal experiences are emphasized in the absence of scientific evidence [82]. Personal narratives can therefore both support and challenge public health communication efforts, depending on their presentation and dissemination. In this review, compared with studies performing sentiment and thematic analyses, relatively few studies examined misinformation or content quality. Nevertheless, available evidence suggests that misinformation and incomplete information are important features of HPV vaccine–related social media communication. This finding is consistent with previous reviews [19,21] and extends the evidence across more social media platforms.

### Platform- and Source-Related Differences in HPV Vaccine Communications

This review also identified differences in HPV vaccine communications by social media platforms and information sources. X was the most studied platform and was commonly used for large-scale text-based analyses. This finding is consistent with previous research showing that X has been widely used in public health research [83]. This pattern may partly reflect X’s text-based structure and the relative accessibility of its data, which have made it particularly amenable to large-scale computational analyses of public health discourse [83]. In contrast, video- or image-based platforms, such as YouTube, TikTok, and Instagram, were examined less often, and studies of these platforms generally relied on manual analysis with smaller sample sizes and focused on uploader characteristics, information quality, content completeness, and user engagement. These differences suggest that platform affordances shape not only how HPV vaccine–related information is communicated but also how researchers can collect, code, and analyze the content [84,85].

Regarding information sources, several studies reported that most HPV vaccine–related content was generated by personal accounts [28,39,45,50,53,59,62,67], with limited contributions from health professionals or governmental agencies. This may reflect the participatory nature of social media, where low barriers to entry allow a wide range of users to share opinions and personal experiences openly [86]. Importantly, content generated by personal accounts was more likely to contain negative sentiments, experiential narratives, and misinformation. In contrast, content produced by health professionals or institutions was generally more positive and of higher quality [34,36,62,66], consistent with the findings of a previous review [17,18]. This suggests that the dominance of personal account-generated content may influence the overall tone and quality of HPV vaccine communications.

Overall, the findings extend previous reviews by showing that HPV vaccine–related social media communication is shaped not only by message content but also by platform environment and information source. This source-platform perspective is important because the same vaccine-related topic, such as safety, effectiveness, or access, may be framed differently depending on whether it is discussed by individuals, health professionals, media accounts, or institutions and whether it appears in text-based, image-based, or video-based social media environments. By synthesizing studies across countries and platforms, this review also suggests that dominant themes in HPV vaccine–related social media discourse may vary by national policy and sociocultural context [46,58,69,71].

### Theoretical and Methodological Implications

Only a few included studies applied established theoretical frameworks to guide analyses. Frameworks such as the HBM, TPB, and EPPM were primarily applied as coding schemes or classification tools [28,33,42-45,54,56,60,63,72]. This finding is consistent with a previous review [20] and further extends the evidence by showing that, specifically in HPV vaccine–related social media research, theoretical frameworks were applied inconsistently and often remained secondary to descriptive or computational analysis. This suggests that the field has become methodologically diverse but remains insufficiently theory-informed. However, because most included studies were designed as content analyses rather than behavioral outcome studies, these frameworks were generally used to classify communication content rather than to test associations with vaccination behavior or other behavioral outcomes.

Regarding analytical approaches, the included studies used various methods, ranging from manual content analysis to automated sentiment analysis, topic modeling, and network analysis. As shown in Figure 4, manual content analysis remained common, particularly for video- or image-based platforms, where communication is often multimodal and requires researchers to consider textual elements alongside visual imagery, audio, speech, and other audiovisual cues [87]. In contrast, automated and machine learning–based approaches were more frequently applied to text-based platforms, particularly X and Weibo, where large-scale textual data were more readily available for computational analysis [30-32,36,38,40,43,55]. This pattern suggests that methodological choices were shaped not only by research questions but also by platform-specific data structures and accessibility. Recent advances in NLP have expanded opportunities to detect, correct, and contextualize medically inaccurate information [22]. However, the application of these methods in HPV vaccine–related social media research remains uneven across platforms. Future research may benefit from combining computational approaches with theory-informed coding frameworks and transparent validation procedures, particularly when analyzing misinformation, emotional content, or multimodal data from short-video platforms.

### Practical Implications

The findings have important practical implications for HPV vaccine communication on social media. First, communication strategies should address not only the benefits of HPV vaccination but also recurring concerns identified across studies [38,46,47,58,64,65,69,71]. Because HPV vaccine–related discourse often combines prevention-oriented information with risk- and concern-oriented messages, public health communication may better reflect online concerns when it provides transparent, balanced, and practical information rather than focusing only on general vaccine promotion [88]. Second, the prominence of personal and experiential narratives suggests that factual information alone may be insufficient in social media environments [39,45,59,64], where narratives can shape health attitudes, intentions, and perceptions of vaccine risk [88]. Public health professionals and institutions may need to develop evidence-based narrative communication that maintains scientific accuracy while remaining understandable, relatable, and responsive to users’ concerns [89]. Such narratives should remain clearly aligned with evidence-based vaccine information [90]. Third, the underrepresentation of authoritative sources in several studies indicates the need to strengthen the visibility and engagement of health professionals, public health institutions, and government agencies on social media [39,49,54,62]. Increasing credible source participation may help counterbalance incomplete or misleading information by improving the visibility of evidence-based health information [91], although the included studies do not provide direct evidence that such participation changes vaccination behavior. Finally, misinformation monitoring should be combined with broader assessments of information completeness, quality, and credibility [92]. Even HPV vaccine–supportive content may be incomplete [51,68,70]. Therefore, interventions should move beyond removing or correcting misinformation alone and also aim to improve the completeness, accessibility, and credibility of HPV vaccine–related information.

### Limitations

This study has some limitations. First, only peer-reviewed English-language papers were included, and studies published in other languages or in gray literature may have been missed. Second, variability in data sources, sampling strategy, search terms, data collection periods, and analytical methods among studies limited the potential for direct comparisons or meta-analyses. Third, studies examining the same platform and overlapping time periods may have captured partially overlapping posts. Therefore, post counts reported in individual studies should not be interpreted as a cumulative total of unique posts. Fourth, most included studies analyzed publicly available social media content and did not measure individual-level exposure, interpretation, vaccination intention, or vaccine uptake. As a result, this review cannot determine the direct behavioral effects of HPV vaccine–related social media content. Finally, platform algorithms, data availability, and API access policies may change over time and may have influenced the scope and reproducibility of the included studies. In addition, changes in platform governance and misinformation moderation policies, particularly during and after the COVID-19 pandemic [93-96], may have influenced the visibility, removal, recommendation, or circulation of HPV vaccine–related content across platforms.

### Conclusions

This scoping review offers a cross-platform, content-focused synthesis of how HPV vaccine–related social media communication has been studied, with attention to communication content, information sources, and analytical approaches. Unlike previous reviews that focused on single platforms, broader HPV-related or cervical cancer–related social media research, or the potential effects of social media on vaccine knowledge and attitudes [17-21], this review clarifies how HPV vaccine–related content has been described, who generates it, and which methods have been used to analyze it. The findings show that HPV vaccine discourse cannot be fully understood through sentiment polarity alone. Instead, it should be interpreted in relation to platform context, information source, narrative forms, misinformation, information quality, and practical barriers. These findings support more theory-informed and platform-sensitive research and may inform communication strategies that strengthen credible, complete, and accessible HPV vaccine information.

## Supplementary material

10.2196/92040 Multimedia Appendix 1 Full database-specific search strategies used in this review.

10.2196/92040 Multimedia Appendix 2 Data extracted in this scoping review.

10.2196/92040 Checklist 1 PRISMA-ScR checklist of this scoping review.

10.2196/92040 Checklist 2 PRISMA-S checklist.

## Abbreviations

BERT: Bidirectional Encoder Representations from Transformers
DMM: Dirichlet multinomial mixture
ELM: elaboration likelihood model
EPPM: extended parallel process model
HBM: health belief model
HPV: human papillomavirus
IMT: information manipulation theory
*JAMA*: *Journal of the American Medical Association*
JBI: Joanna Briggs Institute
LDA: Latent Dirichlet allocation
LIWC: Linguistic Inquiry and Word Count
NLP: natural language processing
PCC: Population-Concept-Context
PRISMA-S: Preferred Reporting Items for Systematic Reviews and Meta-Analyses literature search extension
PRISMA-ScR: Preferred Reporting Items for Systematic Reviews and Meta-Analyses extension for Scoping Reviews
RQ: research question
TPB: theory of planned behavior
WHO: World Health Organization
