# Supplementary material for: Study of Human Papillomavirus Vaccine–Related Social Media Content Across Eight Social Media Platforms: Scoping Review of Communication Content, Information Sources, and Analytical Approaches
Source: J Med Internet Res. 2026 Sep 15;28:e92040. doi: 10.2196/92040 (PMC13577615; doi:10.2196/92040)
Supplement: Supplementary file 2 [file jmir-v28-e92040-s001.docx]

**Search Keywords Details (June 10, 2026)**

**Web of Science (n = 690)**

#1 TS=("HPV vaccine" OR "HPV vaccines" OR "HPV vaccination" OR "papillomavirus vaccine" OR "papillomavirus vaccines" OR "papillomavirus vaccination" OR "human papillomavirus vaccine" OR "human papillomavirus vaccines" OR "human papillomavirus vaccination" OR Gardasil OR "Gardasil 9" OR Cervarix OR Silgard OR "9-valent HPV vaccine" OR "quadrivalent HPV vaccine" OR "bivalent HPV vaccine")

#2 TS=(HPV OR "human papillomavirus" OR "human papilloma virus" OR papillomavirus* OR "cervical cancer" OR "cervical neoplasm" OR "cervical neoplasms" OR "cervical carcinoma" OR "uterine cervical neoplasm" OR "uterine cervical neoplasms" OR "cervical cancer prevention")

#3 TS=(vaccin* OR vaccination* OR immuni* OR immuniz* OR immunis* OR inoculat* OR "vaccine uptake" OR "vaccine acceptance" OR "vaccine hesitancy" OR "vaccine confidence" OR "vaccine refusal" OR "vaccine decision" OR "vaccine decisions")

#4 #1 OR (#2 AND #3)

#5 TS=("social media" OR "social network" OR "social networks" OR "social networking site" OR "social networking sites" OR "social networking service" OR "social networking services" OR SNS OR "online social network" OR "online social networks" OR "online platform" OR "online platforms" OR "digital media" OR "web 2.0" OR "online community" OR "online communities" OR "virtual community" OR "virtual communities" OR "video sharing" OR "video-sharing" OR "video sharing platform" OR "video sharing platforms" OR "video-sharing platform" OR "video-sharing platforms" OR "online video" OR "online videos" OR "short video" OR "short videos" OR "user-generated content" OR "user generated content" OR "online comment" OR "online comments" OR "social media post" OR "social media posts" OR tweet* OR YouTube OR Twitter OR "X Twitter" OR "Twitter/X" OR Instagram OR TikTok OR Facebook OR Weibo OR Bilibili OR Douyin OR Xiaohongshu OR "Little Red Book")

#6 #4 AND #5

**Scopus (n = 820)**

#1 TITLE-ABS-KEY("HPV vaccine" OR "HPV vaccines" OR "HPV vaccination" OR "papillomavirus vaccine" OR "papillomavirus vaccines" OR "papillomavirus vaccination" OR "human papillomavirus vaccine" OR "human papillomavirus vaccines" OR "human papillomavirus vaccination" OR Gardasil OR "Gardasil 9" OR Cervarix OR Silgard OR "9-valent HPV vaccine" OR "quadrivalent HPV vaccine" OR "bivalent HPV vaccine")

#2 TITLE-ABS-KEY( HPV OR "human papillomavirus" OR "human papilloma virus" OR papillomavirus* OR "cervical cancer" OR "cervical neoplasm" OR "cervical neoplasms" OR "cervical carcinoma" OR "uterine cervical neoplasm" OR "uterine cervical neoplasms" OR "cervical cancer prevention")

#3 TITLE-ABS-KEY( vaccin* OR vaccination* OR immuni* OR immuniz* OR immunis* OR inoculat* OR "vaccine uptake" OR "vaccine acceptance" OR "vaccine hesitancy" OR "vaccine confidence" OR "vaccine refusal" OR "vaccine decision" OR "vaccine decisions")

#4 #1 OR (#2 AND #3)

#5 TITLE-ABS-KEY( "social media" OR "social network" OR "social networks" OR "social networking site" OR "social networking sites" OR "social networking service" OR "social networking services" OR SNS OR "online social network" OR "online social networks" OR "online platform" OR "online platforms" OR "digital media" OR "web 2.0" OR "virtual community" OR "virtual communities" OR "video sharing" OR "video-sharing" OR "video sharing platform" OR "video sharing platforms" OR "video-sharing platform" OR "video-sharing platforms" OR "online video" OR "online videos" OR "short video" OR "short videos" OR "user-generated content" OR "user generated content" OR "social media post" OR "social media posts" OR tweet* OR YouTube OR Twitter OR "X Twitter" OR "Twitter/X" OR Instagram OR TikTok OR Facebook OR Weibo OR Bilibili OR Douyin OR Xiaohongshu OR "Little Red Book")

#6 #4 AND #5

**PubMed (n = 702)**

#1 "Papillomavirus Vaccines"[MeSH Terms] OR "HPV vaccine"[Title/Abstract] OR "HPV vaccines"[Title/Abstract] OR "HPV vaccination"[Title/Abstract] OR "papillomavirus vaccine"[Title/Abstract] OR "papillomavirus vaccines"[Title/Abstract] OR "papillomavirus vaccination"[Title/Abstract] OR "human papillomavirus vaccine"[Title/Abstract] OR "human papillomavirus vaccines"[Title/Abstract] OR "human papillomavirus vaccination"[Title/Abstract] OR Gardasil[Title/Abstract] OR "Gardasil 9"[Title/Abstract] OR Cervarix[Title/Abstract] OR Silgard[Title/Abstract] OR "9-valent HPV vaccine"[Title/Abstract] OR "quadrivalent HPV vaccine"[Title/Abstract] OR "bivalent HPV vaccine"[Title/Abstract]

#2 "Papillomavirus Infections"[MeSH Terms] OR "Uterine Cervical Neoplasms"[MeSH Terms] OR HPV[Title/Abstract] OR "human papillomavirus"[Title/Abstract] OR "human papilloma virus"[Title/Abstract] OR papillomavirus*[Title/Abstract] OR "cervical cancer"[Title/Abstract] OR "cervical neoplasm"[Title/Abstract] OR "cervical neoplasms"[Title/Abstract] OR "cervical carcinoma"[Title/Abstract] OR "uterine cervical neoplasm"[Title/Abstract] OR "uterine cervical neoplasms"[Title/Abstract] OR "cervical cancer prevention"[Title/Abstract]

#3 "Vaccination"[MeSH Terms] OR "Immunization"[MeSH Terms] OR vaccin*[Title/Abstract] OR vaccination*[Title/Abstract] OR immuni*[Title/Abstract] OR immuniz*[Title/Abstract] OR immunis*[Title/Abstract] OR inoculat*[Title/Abstract] OR "vaccine uptake"[Title/Abstract] OR "vaccine acceptance"[Title/Abstract] OR "vaccine hesitancy"[Title/Abstract] OR "vaccine confidence"[Title/Abstract] OR "vaccine refusal"[Title/Abstract] OR "vaccine decision"[Title/Abstract] OR "vaccine decisions"[Title/Abstract]

#4 #1 OR (#2 AND #3)

#5 "Social Media"[MeSH Terms] OR "Internet"[MeSH Terms] OR "Health Communication"[MeSH Terms] OR "social media"[Title/Abstract] OR "social network"[Title/Abstract] OR "social networks"[Title/Abstract] OR "social networking site"[Title/Abstract] OR "social networking sites"[Title/Abstract] OR "social networking service"[Title/Abstract] OR "social networking services"[Title/Abstract] OR SNS[Title/Abstract] OR "online social network"[Title/Abstract] OR "online social networks"[Title/Abstract] OR "online platform"[Title/Abstract] OR "online platforms"[Title/Abstract] OR "digital media"[Title/Abstract] OR "web 2.0"[Title/Abstract] OR "virtual community"[Title/Abstract] OR "virtual communities"[Title/Abstract] OR "video sharing"[Title/Abstract] OR "video-sharing"[Title/Abstract] OR "video sharing platform"[Title/Abstract] OR "video sharing platforms"[Title/Abstract] OR "video-sharing platform"[Title/Abstract] OR "video-sharing platforms"[Title/Abstract] OR "online video"[Title/Abstract] OR "online videos"[Title/Abstract] OR "short video"[Title/Abstract] OR "short videos"[Title/Abstract] OR "user-generated content"[Title/Abstract] OR "user generated content"[Title/Abstract] OR "social media post"[Title/Abstract] OR "social media posts"[Title/Abstract] OR tweet*[Title/Abstract] OR YouTube[Title/Abstract] OR Twitter[Title/Abstract] OR "X Twitter"[Title/Abstract] OR "Twitter/X"[Title/Abstract] OR Instagram[Title/Abstract] OR TikTok[Title/Abstract] OR Facebook[Title/Abstract] OR Weibo[Title/Abstract] OR Bilibili[Title/Abstract] OR Douyin[Title/Abstract] OR Xiaohongshu[Title/Abstract]

#6 #4 AND #5

**MEDLINE (n = 624)**

#1 MH "Papillomavirus Vaccines+" OR TI ("HPV vaccine" OR "HPV vaccines" OR "HPV vaccination" OR "papillomavirus vaccine" OR "papillomavirus vaccines" OR "papillomavirus vaccination" OR "human papillomavirus vaccine" OR "human papillomavirus vaccines" OR "human papillomavirus vaccination" OR Gardasil OR "Gardasil 9" OR Cervarix OR Silgard OR "9-valent HPV vaccine" OR "quadrivalent HPV vaccine" OR "bivalent HPV vaccine") OR AB ("HPV vaccine" OR "HPV vaccines" OR "HPV vaccination" OR "papillomavirus vaccine" OR "papillomavirus vaccines" OR "papillomavirus vaccination" OR "human papillomavirus vaccine" OR "human papillomavirus vaccines" OR "human papillomavirus vaccination" OR Gardasil OR "Gardasil 9" OR Cervarix OR Silgard OR "9-valent HPV vaccine" OR "quadrivalent HPV vaccine" OR "bivalent HPV vaccine")

#2 MH "Papillomavirus Infections+" OR MH "Uterine Cervical Neoplasms+" OR TI (HPV OR "human papillomavirus" OR "human papilloma virus" OR papillomavirus* OR "cervical cancer" OR "cervical neoplasm" OR "cervical neoplasms" OR "cervical carcinoma" OR "uterine cervical neoplasm" OR "uterine cervical neoplasms" OR "cervical cancer prevention") OR AB (HPV OR "human papillomavirus" OR "human papilloma virus" OR papillomavirus* OR "cervical cancer" OR "cervical neoplasm" OR "cervical neoplasms" OR "cervical carcinoma" OR "uterine cervical neoplasm" OR "uterine cervical neoplasms" OR "cervical cancer prevention")

#3 MH "Vaccination+" OR MH "Immunization+" OR TI (vaccin* OR vaccination* OR immuni* OR immuniz* OR immunis* OR inoculat* OR "vaccine uptake" OR "vaccine acceptance" OR "vaccine hesitancy" OR "vaccine confidence" OR "vaccine refusal" OR "vaccine decision" OR "vaccine decisions") OR AB (vaccin* OR vaccination* OR immuni* OR immuniz* OR immunis* OR inoculat* OR "vaccine uptake" OR "vaccine acceptance" OR "vaccine hesitancy" OR "vaccine confidence" OR "vaccine refusal" OR "vaccine decision" OR "vaccine decisions")

#4 #1 OR (#2 AND #3)

#5

MH "Social Media+" OR MH "Internet+" OR MH "Health Communication+" OR TI ("social media" OR "social network" OR "social networks" OR "social networking site" OR "social networking sites" OR "social networking service" OR "social networking services" OR SNS OR "online social network" OR "online social networks" OR "online platform" OR "online platforms" OR "digital media" OR "web 2.0" OR "virtual community" OR "virtual communities" OR "video sharing" OR "video-sharing" OR "video sharing platform" OR "video sharing platforms" OR "video-sharing platform" OR "video-sharing platforms" OR "online video" OR "online videos" OR "short video" OR "short videos" OR "user-generated content" OR "user generated content" OR "social media post" OR "social media posts" OR tweet* OR YouTube OR Twitter OR "X Twitter" OR "Twitter/X" OR Instagram OR TikTok OR Facebook OR Weibo OR Bilibili OR Douyin OR Xiaohongshu OR "Little Red Book") OR AB ("social media" OR "social network" OR "social networks" OR "social networking site" OR "social networking sites" OR "social networking service" OR "social networking services" OR SNS OR "online social network" OR "online social networks" OR "online platform" OR "online platforms" OR "digital media" OR "web 2.0" OR "virtual community" OR "virtual communities" OR "video sharing" OR "video-sharing" OR "video sharing platform" OR "video sharing platforms" OR "video-sharing platform" OR "video-sharing platforms" OR "online video" OR "online videos" OR "short video" OR "short videos" OR "user-generated content" OR "user generated content" OR "social media post" OR "social media posts" OR tweet* OR YouTube OR Twitter OR "X Twitter" OR "Twitter/X" OR Instagram OR TikTok OR Facebook OR Weibo OR Bilibili OR Douyin OR Xiaohongshu OR "Little Red Book")

#6 #4 AND #5

**Embase (n = 2,623)**

#1 'papillomavirus vaccine'/exp OR 'human papillomavirus vaccine'/exp OR 'HPV vaccine':ti,ab,kw OR 'HPV vaccines':ti,ab,kw OR 'HPV vaccination':ti,ab,kw OR 'papillomavirus vaccine':ti,ab,kw OR 'papillomavirus vaccines':ti,ab,kw OR 'papillomavirus vaccination':ti,ab,kw OR 'human papillomavirus vaccine':ti,ab,kw OR 'human papillomavirus vaccines':ti,ab,kw OR 'human papillomavirus vaccination':ti,ab,kw OR Gardasil:ti,ab,kw OR 'Gardasil 9':ti,ab,kw OR Cervarix:ti,ab,kw OR Silgard:ti,ab,kw

#2 'papillomavirus infection'/exp OR 'human papillomavirus infection'/exp OR 'cervix cancer'/exp OR 'uterine cervix cancer'/exp OR HPV:ti,ab,kw OR 'human papillomavirus':ti,ab,kw OR 'human papilloma virus':ti,ab,kw OR papillomavirus*:ti,ab,kw OR 'cervical cancer':ti,ab,kw OR 'cervical neoplasm':ti,ab,kw OR 'cervical neoplasms':ti,ab,kw OR 'cervical carcinoma':ti,ab,kw OR 'uterine cervical neoplasm':ti,ab,kw OR 'uterine cervical neoplasms':ti,ab,kw OR 'cervical cancer prevention':ti,ab,kw

#3 'vaccination'/exp OR 'immunization'/exp OR vaccin*:ti,ab,kw OR vaccination*:ti,ab,kw OR immuni*:ti,ab,kw OR immuniz*:ti,ab,kw OR immunis*:ti,ab,kw OR inoculat*:ti,ab,kw OR 'vaccine uptake':ti,ab,kw OR 'vaccine acceptance':ti,ab,kw OR 'vaccine hesitancy':ti,ab,kw OR 'vaccine confidence':ti,ab,kw OR 'vaccine refusal':ti,ab,kw OR 'vaccine decision':ti,ab,kw OR 'vaccine decisions':ti,ab,kw

#4 #1 OR (#2 AND #3)

#5 'social media'/exp OR 'social network'/exp OR 'internet'/exp OR 'health communication'/exp OR 'mass communication'/exp OR 'social media':ti,ab,kw OR 'social network':ti,ab,kw OR 'social networks':ti,ab,kw OR 'social networking site':ti,ab,kw OR 'social networking sites':ti,ab,kw OR 'social networking service':ti,ab,kw OR 'social networking services':ti,ab,kw OR SNS:ti,ab,kw OR 'online social network':ti,ab,kw OR 'online social networks':ti,ab,kw OR 'online platform':ti,ab,kw OR 'online platforms':ti,ab,kw OR 'virtual community':ti,ab,kw OR 'virtual communities':ti,ab,kw OR 'video sharing':ti,ab,kw OR 'video-sharing':ti,ab,kw OR 'video sharing platform':ti,ab,kw OR 'video sharing platforms':ti,ab,kw OR 'video-sharing platform':ti,ab,kw OR 'video-sharing platforms':ti,ab,kw OR 'online video':ti,ab,kw OR 'online videos':ti,ab,kw OR 'short video':ti,ab,kw OR 'short videos':ti,ab,kw OR 'user-generated content':ti,ab,kw OR 'user generated content':ti,ab,kw OR 'social media post':ti,ab,kw OR 'social media posts':ti,ab,kw OR tweet*:ti,ab,kw OR YouTube:ti,ab,kw OR Twitter:ti,ab,kw OR 'X Twitter':ti,ab,kw OR 'Twitter/X':ti,ab,kw OR Instagram:ti,ab,kw OR TikTok:ti,ab,kw OR Facebook:ti,ab,kw OR Weibo:ti,ab,kw OR Bilibili:ti,ab,kw OR Douyin:ti,ab,kw OR Xiaohongshu:ti,ab,kw OR 'Little Red Book':ti,ab,kw

#6 #4 AND #5
